# Cancer Vaccines: The Next Generation of Tools to Monitor the Anticancer Immune Response

**DOI:** 10.1371/journal.pmed.0020339

**Published:** 2005-10-25

**Authors:** Frank O Nestle, Giulia Tonel, Arpad Farkas

## Abstract

Nestle and colleagues discuss a new immunomonitoring tool, described by Chen et al. in *PLoS Medicine,* that may give insight into the vaccine-induced anticancer immune response.

The promise of a cancer vaccination to fight tumors using the patient's own immune system is at the same time fascinating and challenging. Since cancer typically strikes at an older age, there is no evolutionary pressure on the immune system to develop successful anticancer effectors. This lack of evolutionary pressure to fight cancer is an apparent contrast to infectious diseases, for which a reasonable argument can be made that the immune system has evolved to fight pathogens. These assumptions might explain the relative success of vaccine development in the area of infectious diseases, and the long history of failed attempts in cancer vaccination.

And yet there is overwhelming evidence from laboratory studies that specific effectors of the immune system are able to recognize and destroy cancer cells. Soluble effectors of the immune system, such as antibodies, are now reliable weapons in the fight against lymphomas [1]. The graft-versus-leukemia effect (immunological rejection of leukemia cells following bone marrow transplantation) strongly indicates the power of the immune system to control certain tumors [2]. Thus, the immune system, even though primarily evolved to fight pathogens, clearly has the means to fight cancer.

## Challenges of Cancer Vaccination

What are the prerequisites for a successful anticancer immune response? Before destruction of a tumor is achieved by the host immune system, a simplified four-step scenario has to take place: (1) activation and expansion of tumor-specific effector cells, (2) migration of effector cells to the tumor site, (3) effector-cell recognition of tumor cells, and (4) effector-cell destruction of tumor cells. In addition, a quantitative balance between the number of tumor cells and effector cells needed to destroy the tumor has to be taken into account. All these steps need to be investigated and controlled for successful cancer vaccination.

Cytotoxic T cells are among the best-investigated effector cells of the immune system. There is strong evidence that the presence of intratumoral T cells correlates with improved clinical outcome in certain human cancers during the natural immune response against tumors [3]. Dendritic cells are among the most powerful activators of tumor-specific helper cells and cytotoxic T cells [4]. Expansion of human tumor antigen-specific helper cells and cytotoxic T cells in peripheral blood has been demonstrated using antigen-pulsed dendritic cells as well as intracutaneous peptide immunization in the presence or absence of adjuvant [5–7].

Specific effectors of the immune system are able to recognize and destroy cancer cells.

There is, however, limited insight into the magnitude, breadth, and molecular nature of the induced immune responses. There are also currently no means to discriminate between protective and nonprotective (curative and noncurative) T cell responses. In fact, we are not certain about the frequency of tumor antigen-specific T cells that is necessary for tumor destruction. While viral protection models suggest that a high frequency (number) of vaccine-induced specific effectors is necessary, alternative hypotheses favor the generation of low-frequency vaccine-induced responses, which might in turn affect pre-existing antitumor-specific T cells [8]. Therefore, new developments in the area of monitoring and understanding the tumor-specific immune response, in combination with small innovative pilot vaccine trials, are needed.

## Monitoring of the Cancer Immune Response

There are many ways to assess a cancer-specific immune response, including monitoring (1) direct cytotoxicity of effectors, as measured by chromium release assays (see Glossary), (2) cytokine release from effector cells, as assessed by flow cytometry or enzyme-linked immunosorbent assay techniques, (3) T cell receptor (TCR) specificities, as assessed by MHC-peptide multimers, (4) clonal composition of the T cell response via CDR (complementarity-determining region) 3 spectratyping, and (5) T cell degranulation via cell surface exposure of cluster designation (CD)107 [9–11].

**Glossary**

**Cluster designation (CD) 107:** A lysomal membrane protein that translocates to the cell surface during the killing process.

**CDR3 spectratyping:** CDRs of TCRs are the parts of these molecules that determine their specificity and make contact with specific ligands. Spectratyping defines certain types of DNA gene segments that constitute the CDR.

**Chromium release assay:** Assay for cytotoxic activity of killer cells.

**Enzyme-linked immunosorbent assay (ELISA):** A serological assay in which a bound antigen or antibody is detected by a linked enzyme that converts a colorless substrate into a colored product.

**Flow cytometry:** Analysis of biological material by detection of light-absorbing or fluorescing properties of cells.

**HLA-A2-Immunoglobulin dimers:** Dimers of human leukocyte antigen domains fused to an immunoglobulin scaffold.

**MHC-peptide multimers:** MHC-peptide multimers detect vaccine-specific T cells.

**Multiparametric microarray platform:** Platform to assess multiple parameters at once on a small glass chip.

**NK cells:** NK cells are large, granular non-T, non-B lymphocytes that kill certain tumor cells.

**NKT cells:** NKT cells are lymphocytes that share features of both T cells and NK cells.

**TCR specificities:** Specific TCRs recognizing MHC-peptide complexes.

Many of these techniques are useful, but fail to fully assess the functional complexity of an anticancer T cell response in a comprehensive manner. Analyzing T cell specificities using MHC-peptide multimers has revolutionized the field of cancer vaccination, but does not provide insights into T cell function. Detection of interferon-γ production by tumor-specific T cells gives some functional insights, but lacks information about TCR specificities and does not cover the broad spectrum of potential effector cytokines. Indeed, studies of chronic viral infection models suggest the relevance of multiple cytokines such as interferon γ, interleukin 2, and tumor necrosis factor α for effector T cell fitness [12].

## A New Study of a Novel Immunomonitoring Tool

A new study by Chen et al. in the current issue of *PLoS Medicine* provides a novel approach to these scientific issues, and increases our insight into the vaccine-induced anticancer immune response using a novel immunomonitoring tool [13]. The tool combines analysis of TCR specificities with detection of cytokine production in a multiparametric microarray platform. The authors array HLA-A2-immunoglobulin dimers loaded with the peptide of interest with cytokine-capturing antibodies on three-dimensional substrates composed of microscope slides coated with a polyacrylamide gel. This allows the comprehensive analysis of multiple T cell specificities and functional outcomes. Similar platforms were recently used to investigate antigen-specific T cell clones [14]. The current study, however, provides the first comprehensive analysis of vaccine-induced antitumor immune responses in patients with cancer.

One of the most striking findings is the marked variation of responses toward well-defined peptide vaccines. Variation of the response was seen both at the level of a single patient and, independently, at the level of the specific antigen. Thus, no patient or antigen-specific functional response pattern was observed. Even though the current study does not allow definitive conclusions about a link between specific cytokine secretion profiles and clinical outcomes, it appears as if both interferon γ and tumor necrosis factor α were relevant for tumor clearance, as indicated by prolonged recurrence-free periods in patients with such a cytokine profile.

## Implications of the Study

The study raises several questions. Are cytokine signatures present in certain subpopulations of effector T cells, especially those successful in tumor rejection? Are cytokine signatures predictive of the clinical outcome? It will be interesting to test T cell subpopulations, especially those derived from secondary lymphoid tissues and the tumor site. Are there cytokine signatures in response to pathogens or pathogen-specific vaccines, and how do these signatures differ from those induced by cancer vaccines? Antibody responses might be detected by protein microarrays [15]. Are there ways to array and functionally analyze other components of the immune system such as NKT cells, NK cells, or granulocytes?

Taken together, the current study establishes the fundamentals for future application of high-throughput multiparametric platforms that simultaneously capture antigen-specific T cells and detect secreted products in the analysis of tumor and, potentially, pathogen-specific immune responses.

## Abbreviations

CDR: complementarity-determining region
MHC: major histocompatibility complex
NK: natural killer
TCR: T cell receptor
